# Construction of the metabolic reprogramming-associated gene signature for clear cell renal cell carcinoma prognosis prediction

**DOI:** 10.1186/s12894-023-01317-3

**Published:** 2023-09-15

**Authors:** Rongfen Tai, Jinjun Leng, Wei Li, Yuerong Wu, Junfeng Yang

**Affiliations:** 1 State Key Laboratory of Primate Biomedical Research, Institute of Primate Translational Medicine, Kunming University of Science and Technology, Kunming, Yunnan 650500 China; 2 Department of Urology, The First People’s Hospital of Yunnan Province, The Affiliated Hospital of Kunming University of Science and Technology, Kunming, Yunnan 650032 China

**Keywords:** Metabolic reprogramming, Gene signature, Clear cell renal cell carcinoma, Prognosis, Survival predicting

## Abstract

**Background:**

Metabolism reprogramming is a hallmark that associates tumor growth, metastasis, progressive, and poor prognosis. However, the metabolism-related molecular patterns and mechanism in clear cell renal cell carcinoma (ccRCC) remain unclear. Herein, the purpose of this study was to identify metabolism-related molecular pattern and to investigate the characteristics and prognostic values of the metabolism-related clustering.

**Methods:**

We comprehensively analyzed the differentially expressed genes (DEGs), and metabolism-related genes (MAGs) in ccRCC based on the TCGA database. Consensus clustering was used to construct a metabolism-related molecular pattern. Then, the biological function, molecular characteristics, Estimate/immune/stomal scores, immune cell infiltration, response to immunotherapy, and chemotherapy were analyzed. We also identified the DEGs between subclusters and constructed a poor signature and risk model based on LASSO regression cox analysis and univariable and multivariable cox regression analyses. Then, a predictive nomogram was constructed and validated by calibration curves.

**Results:**

A total of 1942 DEGs (1004 upregulated and 838 downregulated) between ccRCC tumor and normal samples were identified, and 254 MRGs were screened out from those DEGs. Then, 526 ccRCC patients were divided into two subclusters. The 7 metabolism-related pathways enriched in cluster 2. And cluster 2 with high Estimate/immune/stomal scores and poor survival. While, cluster 1 with higher immune cell infiltrating, expression of the immune checkpoint, IFN, HLA, immune activation-related genes, response to anti-CTLA4 treatment, and chemotherapy. Moreover, we identified 295 DEGs between two metabolism-related subclusters and constructed a 15-gene signature and 9 risk factors. Then, a risk score was calculated and the patients into high- and low-risk groups in TCGA-KIRC and E-MTAB-1980 datasets. And the prediction viability of the risk score was validated by ROC curves. Finally, the clinicopathological characteristics (age and stage), risk score, and molecular clustering, were identified as independent prognostic variables, and were used to construct a nomogram for 1-, 3-, 5-year overall survival predicting. The calibration curves were used to verify the performance of the predicted ability of the nomogram.

**Conclusion:**

Our finding identified two metabolism-related molecular subclusters for ccRCC, which facilitates the estimation of response to immunotherapy and chemotherapy, and prognosis after treatment.

**Supplementary Information:**

The online version contains supplementary material available at 10.1186/s12894-023-01317-3.

## Introduction

Renal cell carcinoma (RCC) is a malignant solid tumor that accounts for 2.2% of the total cancer cases and 1.8% of the cancer deaths in 2020 [1]. In the United States, there are an estimated 79,000 new cases and 13,920 deaths in 2022 [2]. Clear cell RCC (ccRCC) is the most common subtype of RCC among approximately 80% of kidney cancers [3]. CcRCC is histologically characterized by clear cytoplasm, with nested clusters of cells surrounded by a dense endothelial network [4]. In the past decade, the survival period of ccRCC patients has been prolonged [5], however, approximately 30% of ccRCC patients develop recurrence and metastasis after surgical resection [6]. Cytokine is still a treatment option for advanced ccRCC [7], and targeted therapies are the current treatment options for ccRCC, such as tyrosine kinase inhibitors (TKIs) [8]. The use of inhibitors to curb the overexpression of immune checkpoint ligands and the immunomodulatory effects of anti-angiogenic agents were current standard of metastatic RCC care [9]. And risk stratification exerts a prominent role in clinical trial design and treatment selection in ccRCC [10, 11]. Therefore, developing a new prognostic risk model for ccRCC is crucial for designing therapeutic options.

Metabolic reprogramming is a cancer hallmark that supports tumor cell proliferation and growth in nutrient-poor settings [12]. There are different from normal cells, tumor cells maintain their survival and growth under the normal or even under severe microenvironments through energy acquisition and biomass synthesis by reprogramming catabolic and anabolic metabolism [13]. Warburg effects or aerobic glycolysis is a core metabolic process to generate energy and provide essential glycolytic intermediates [14]. In addition, glutaminolysis and fatty acid oxidation are the other core metabolic processes of catabolic and anabolic processes, such as protein, nucleotide biosynthesis, one-carbon metabolism, and lipid biosynthesis [15]. Recent pieces of evidence have found that cancer metabolism affects the proliferation, differentiation, execution of effector functions of cancer cells, stromal and immune cells in the tumor microenvironment (TME) to regulate response to antitumor treatment [13, 16, 17]. ccRCC was regarded as a metabolic disease in the sense that many of metabolism reprogramming, including reprogramming of glucose, fatty acid, the tricarboxylic acid cycle (TAC), tryptophan, arginine, and glutamine, has been widely found participated in the development and metastasis [18, 19], that provides new biomarkers, molecular mechanisms, and therapeutic strategies for ccRCC. The inactivation of von Hippel Lindau (VHL) gene was found is almost universal in ccRCC, which leads to the activation of hypoxia-relevant pathway and metabolic pathways such as glycolysis pathway and glutamine reprogramming into antioxidant pathways [20–22]. However, the metabolism-related molecular pattern and characteristics remain unclear.

In the present study, we comprehensively identified metabolism-related molecular patterns, the molecular characteristics of metabolism-related patterns, the landscape of immune cell infiltration, and the prognostic values of metabolism-related genes based on the TCGA-KIRC from The Cancer Genome Atlas (TCGA) database, GSE73731 dataset from Gene Expression Omnibus (GEO), E-MTAB-1980 dataset from ArrayExpress database.

## Methods

### Data collating and processing

In this study, the mRNA expression profiles and corresponding clinical information were obtained from The Cancer Genome Atlas (TCGA, https://portal.gdc.cancer.gov/), which contains a total of 533 KIRC samples and 72 paracancerous samples. According to the data integrality, 526 KIRC samples and 72 paracancerous samples were ultimately involved in the subsequent analysis. Meanwhile, the gene expression profiles GSE73731 dataset which contained 265 ccRCC samples were obtained from Gene Expression Omnibus (GEO, https://www.ncbi.nlm.nih.gov/geo/), performed on Affymetrix Human Genome U133 Plus 2.0 Array. And the accession number of ArrayExpress is E-MTAB-1980, including 101 who had follow-up information obtained from the ArrayExpress database (https://www.ebi.ac.uk/arrayexpress).

### Screening the differentially expressed genes (DEGs) in ccRCC

Limma package in R was performed to identify the DEGs from 526 KIRC samples and 72 paracancerous samples, which included upregulated and downregulated DEGs according to the false discovery rate (FDR) < 0.5 and log_2_ |fold change (FC)| > 1. The results were visualized by the ggplot2 R package. Then, a total of 254 metabolic-associated genes (MAGs) expressions were screened and visualized by pheatmap R package (Table S1).

### Construction of the MAG signature

After combining the expression of MAGs with survival time, then, the prognostic MAGs were identified by univariable Cox analysis using the Survival package in R.

### Development of metabolic-related subclusters using consensus clustering

Unsupervised hierarchical clustering was performed using the ConsensusClusterPlus R package to group the prognostic MAGs in the TCGA database. The optimal number of clusters (k value) was determined according to the cumulative distribution function (CDF) reached an approximate maximum. Besides, t-Distributed Stochastic Neighbor Embedding (t-SNE), which is a non-linear dimensionality reduction method, was performed to detect the accuracy of clustering. Meanwhile, the unsupervised hierarchical clustering was also performed using the ConsensusClusterPlus R package and validated using t-SNE in the GSE73731 dataset. Finally, the Subnetwork Mappings in Alignment of Pathways (SubMAP) matrix was conducted to investigate the similarity of a subset from TCGA and GEO datasets.

### Gene set enrichment analysis (GSEA)

GSEA was performed to explore the potential molecular mechanism between the two clusters based on Hallmark gene sets from Molecular Signature Database (MSigDB, https://www.gsea-msigdb.org/gsea/msigdb/).

### Estimation of the tumor microenvironment (TME) cell infiltration

The immune score, stromal score, and ESTIMATE score of each sample were estimated using the estimate package in R, and the differences between the two clusters were determined using the Wilcoxon rank-sum test.

### Estimation of the immune cell landscape

The immune cell fractions were identified using CIBERSORT, which is a deconvolution algorithm based on the expression of 547 genes. And single-sample gene set enrichment (ssGSEA) algorithm was performed using the GSVA package in R to quantize the relative abundance of each immune cell type.

### Analysis of the core biological pathways of ccRCC

Gene set variation analysis (GSVA) algorithm was used to explore the distinct signaling pathways between subclusters based on the gene expression profiles. The gene sets associated with TME-related pathways were downloaded from The Molecular Signatures Database v7.2 (MSigDB, https://www.gsea-msigdb.org/gsea/msigdb/). The enrichment score of pathways in each sample was calculated and the differences between subclusters were detected using the Wilcoxon rank-sum test. The differential pathways were screened with the criteria of FDR < 0.05 and |log2 (FC)| > 0.2.

### Prediction of immunotherapy response between clusters

Tumor immune dysfunction and exclusion (TIDE, http://tide.dfci.harvard.edu/login/) and the SubMap algorithm were used to predict the likelihood of response to immunotherapy. Human leukocyte antigen (HLA) genes and immune checkpoints exert crucial roles in response to immunotherapy. The differences in TIDE scores and differential pathways were determined by Wilcoxon rank-sum test.

### Prediction of drug sensitivity between clusters

The sensitivity of each sample to chemotherapy drugs was decided by Genomics of Drug Sensitivity in Cancer (GDSC, https://www.cancerrxgene.org/). The half-maximal inhibitory concentration (IC50) value was assessed by ride regression using the pRRophetic R package. The smaller the IC50 value indicated the stronger inhibitory effects on cancer cells.

### Construction of the risk signature and model

The DEGs between subclusters were obtained using the Limma package in R with the criteria of FDR < 0.05 and |log2 (FC)| > 1. Then, the prognostic associated DEGs were screened using univariable cox regression analysis in the TCGA database. Hazard ration (HR) > 1 indicated the poor survival outcomes, while HR < 1 indicated the good survival outcomes. Gene with *P*-value < 0.05 were identified as prognostic associated DEGs. The least absolute shrinkage and selection operation (LASSO) Cox regression analysis was used to identify risk gene signature based on prognostic associated DEGs using the glmnet package in R. Then, the multivariable cox regression analysis was used to assess the independence of the risk gene signature via the coxph package in R. According to the risk gene signature, the risk score was calculated as the following formula, risk score = $\sum _{i=1}^{n}coef \left(genei\right)*expr \left(genei\right)$, coef represented the risk coefficient, and expr represented the expression of each gene. Patients were divided into high-risk and low-risk groups based on the median risk score. Kaplan-Meier curves were used to compare the differences in overall survival (OS) between the two groups. Time-dependent receiver operating characteristic curves (ROC) for 1-, 3-, 5-years OS were used to predict the predictive power of the risk model. In addition, the survival information obtained from the ArrayExpress database was used to validate the risk model.

### Construction of predictive nomogram of ccRCC patients

The clinicopathological factors and risk score incorporated into the nomogram to construct the predictive model for prognosis using the rms package in R. Calibration curves were established to evaluate the predictive accuracy of the nomogram.

### Statistical analysis

In this study, all statistical analyses and visualized were performed using R software version 3.4.4 according to previous manuscript from Assel et al. [20]. The continuous variables were shown as mean ± standard deviation (SD), Chi-square test was used to analysis the significance of difference of the categorical variables. And survival analysis was performed using Kaplan-Meier plots and log-rank tests. p-value < 0.05 was considered statistical significance.

## Results

### Identification of the prognostic associated metabolism-related genes (MRGs)

**Fig. 1:**
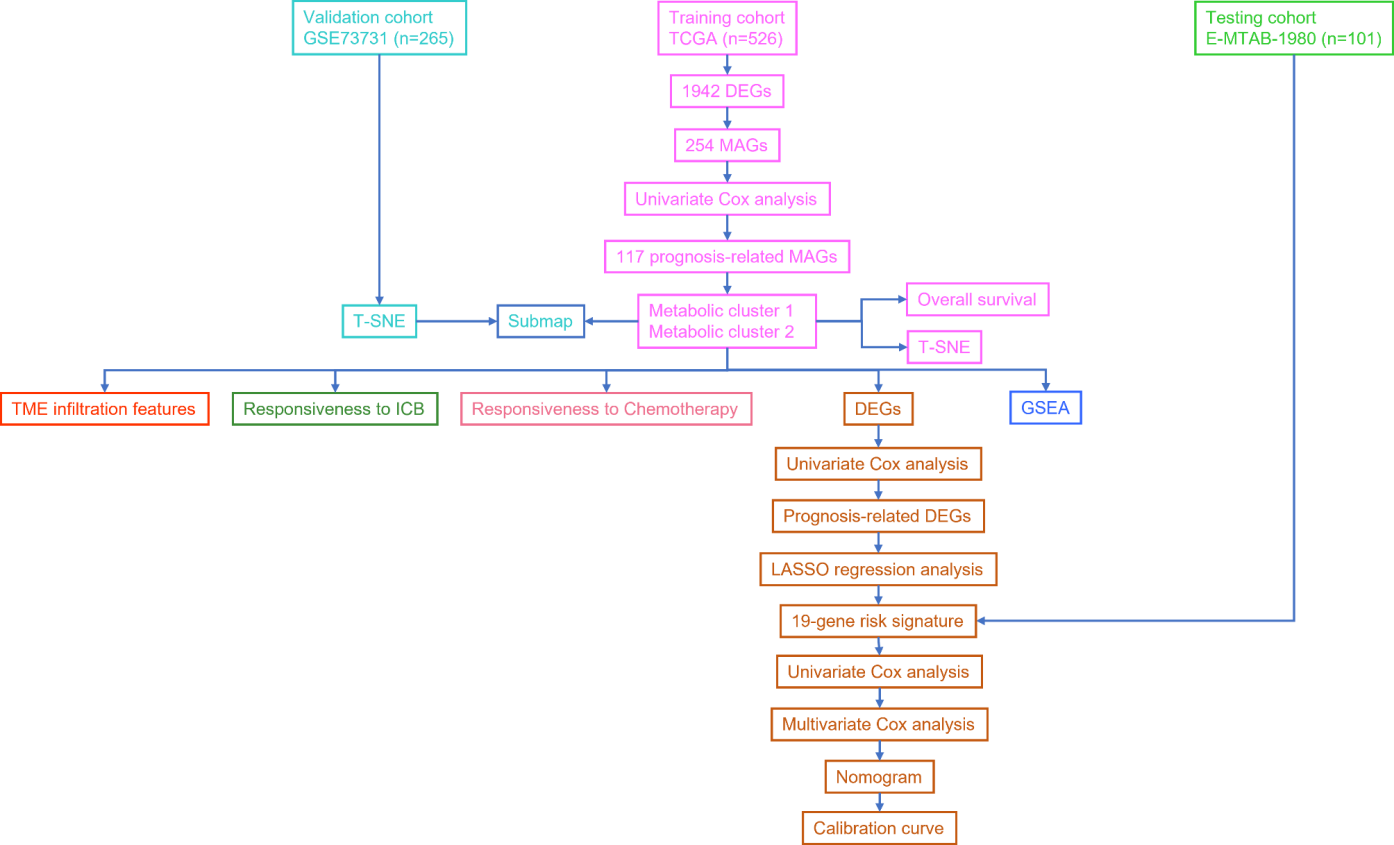
Workflow chart of this study

**Fig. 2:**
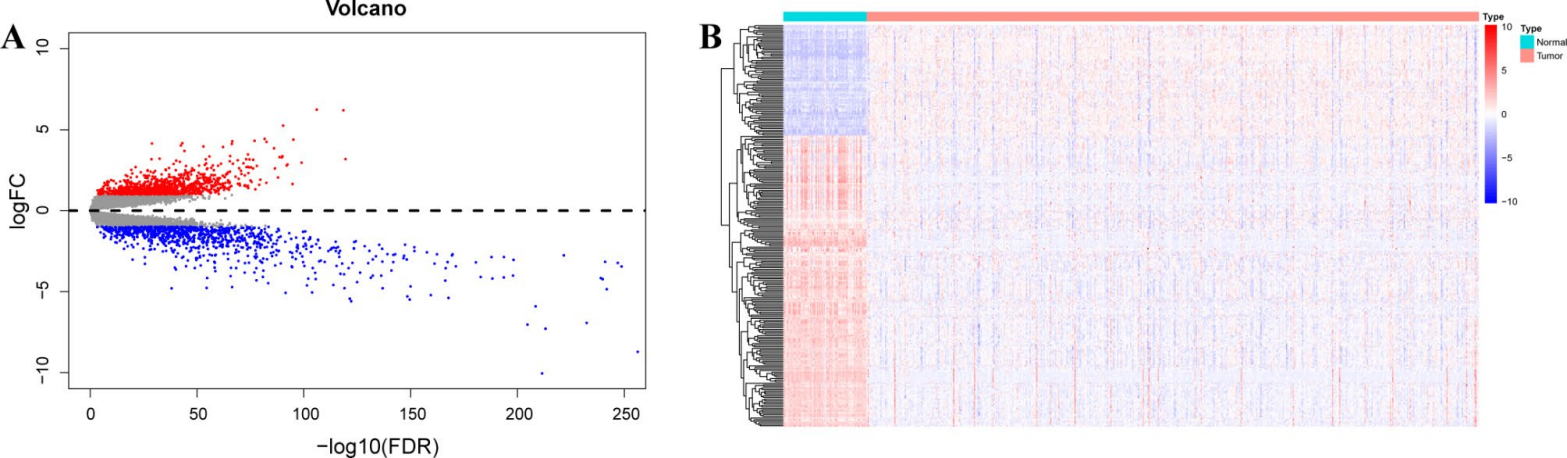
**Identification of the prognostic associated metabolism-related genes (MRGs).** (**A**) Volcano plot showing the differentially expressed genes between KIRC samples and paracancerous samples. (**B**) Heatmap showing the differential expression of a total of 254 metabolic-related genes (MRGs)

The design of this study was shown in Fig. 1. 526 KIRC samples and 72 paracancerous samples from the TCGA database. A total of 1942 DEGs, including 1004 upregulated and 838 downregulated DEGs, were identified between tumor and normal samples in ccRCC (Fig. 2A, Table S2). Then, 254 MRGs were screened between tumor and normal samples in ccRCC (Fig. 2B).

### Construction of metabolism-related subclusters for ccRCC

**Fig. 3:**
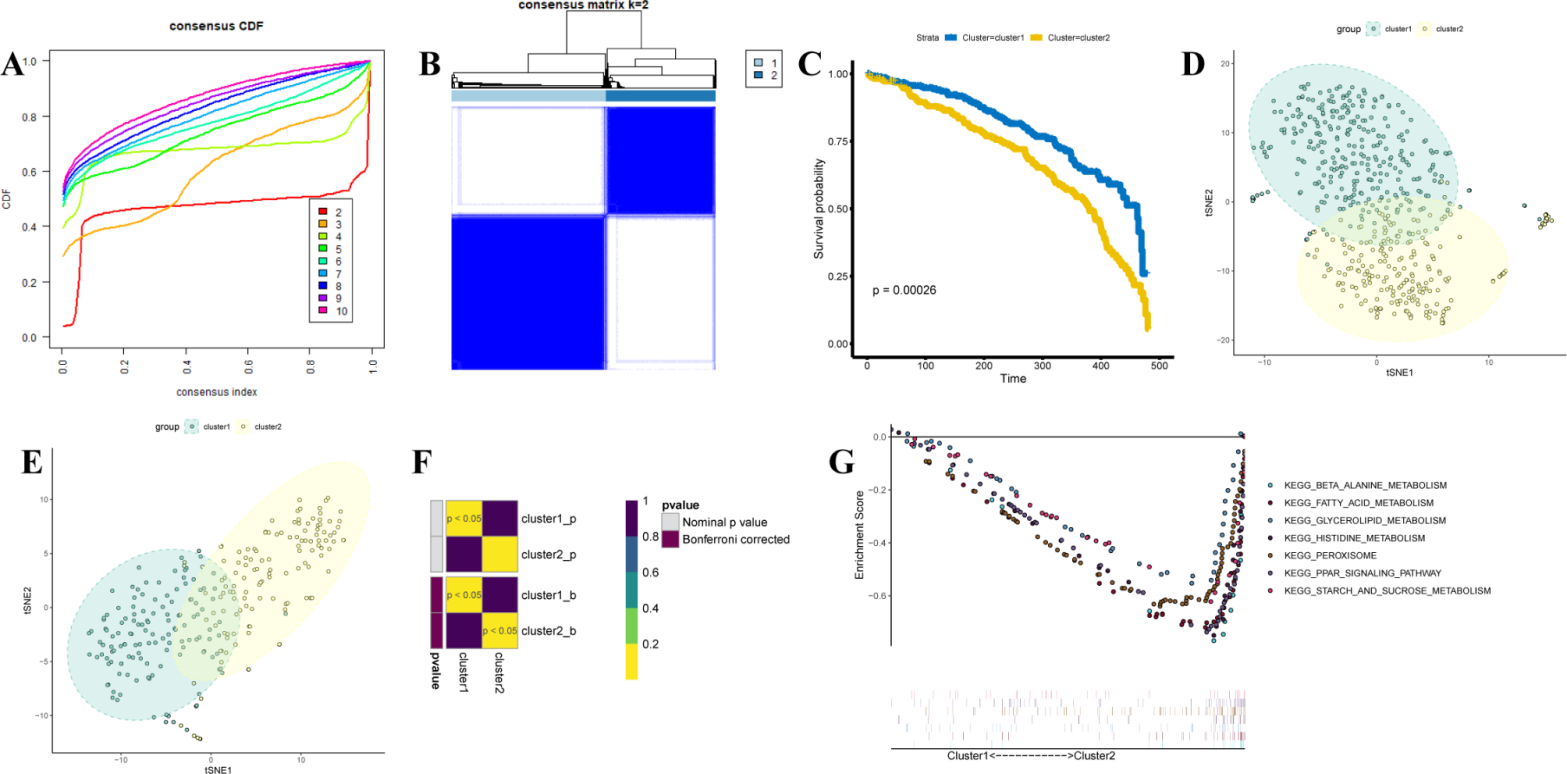
**Construction of metabolism-related subclusters for ccRCC.** (**A**) The CDF curve for k = 2 to 10. (**B**) The consensus clustering matrix at k = 2. (**C**) Kaplan-Meier overall survival curves of the three clusters. (**D**) The t-SNE scatter plots show the classification into two ccRCC molecular subtypes based on the gene expression profiles in the TCGA database. (**E**) The t-SNE scatter plots show the classification into two ccRCC molecular subtypes based on the gene expression profiles in the GEO database (GSE73731 dataset). (**F**) Submap showing the similarity of two ccRCC molecular subtypes between TCGA and GEO databases. (**G**) GSEA has shown metabolism-related pathways enriched in cluster 2

We further incorporated the survival data and MRGs into a univariable cox regression model to identify the prognostic related genes. And the results showed that 117 MRGs were associated with ccRCC prognosis (Table 1). Then, 526 ccRCC patients were divided into two distinct subclusters using consensus clustering, including cluster 1 (n = 217) and cluster 2 (n = 309) (Fig. 3A-B, Figure S1, Table 2). OS curve indicated the patients in cluster 1 with a better prognosis than those in cluster 2 (Fig. 3C). The robustness of the classification was verified by t-SNE methods, and we observed the discrimination of two subclusters (Fig. 3D). In addition, we also classified ccRCC patients into subclusters using unsupervised consensus clustering in the testing cohort (GSE73731 dataset), and the results showed the discrimination between two subclusters (Figure S2). The dimensional reduction also showed the discrimination of subclusters in the testing cohort (Fig. 3E). Submap was used to compare subclusters in training and testing cohorts, as shown in Fig. 3F, the strong similarity between subclusters in training and testing cohorts. Considering the classification in ccRCC patients based on MRGs, we investigated whether relevant signaling pathways varied between two subclusters. GSEA results showed that cluster 2 is associated with beta-alanine metabolism, fatty acid metabolism, glycerolipid metabolism, histidine metabolism, peroxisome, PPAR signaling pathway, and starch and sucrose metabolism (Fig. 3G).

**Table 1:** Univariable Cox analysis of the survival associated metabolic-related genes (GRGs)

Gene	P-value
GSTM3	0.001417
FECH	0.000174
DBT	1.00E-08
HADH	1.00E-08
AK3	0.004502
FMO5	0.006443
COLGALT1	2.10E-06
NNMT	0.011051
CROT	0.010349
TYMP	0.004195
ATP6V1C2	0
HSD11B2	0.000655
ENO2	0.005066
FDX1	0.005869
ALDH6A1	0
ENPP3	9.27E-05
DEGS2	0.008759
TYMS	0.005912
ATP6V1H	0.009395
ACAA1	0.048444
GPAT3	2.08E-05
SUCLG2	0.000698
ACADSB	1.40E-07
ABAT	0.000805
GPD1L	1.32E-05
GAPDH	0.009232
GLDC	0.009796
UQCRFS1	0.017291
PFKP	0.000214
NDUFS1	4.00E-08
PIP5K1B	0.013496
BCKDHB	0.000831
ALAD	1.81E-05
LDHD	3.80E-06
CHST11	0.018878
PCCA	0
L2HGDH	2.05E-05
CYP39A1	0.014135
CYP2J2	0.005771
HOGA1	0.044751
ATP6V1A	1.10E-07
GCNT4	0.000632
ACADM	0
SDHD	0.000205
CDS1	2.80E-07
OXCT1	0.005714
HMGCS2	0.000111
CAT	1.30E-07
AUH	9.40E-07
NNT	9.40E-07
SUCLA2	0
HIBADH	0.005361
OGDHL	5.50E-07
ALDOB	2.19E-05
SCD5	5.52E-05
DLD	0.000139
HSD3B7	0.000374
ACSM3	0.003205
ADA	0.000266
CA2	0.006295
FBP1	9.39E-06
DAO	9.21E-05
PLCB2	0.00596
GALM	0.002137
PFKM	0.006945
PSAT1	1.22E-06
TCIRG1	1.00E-08
PANK1	0
FABP5	0.000341
SLC22A13	0.0005
IL4I1	0.024682
FMO4	0.00245
PCK2	0.006542
HIBCH	5.60E-07
ACLY	5.55E-05
ALDH5A1	0.032277
RIMKLA	1.23E-06
ALOX5	0.007044
TREH	0.000136
RRM2	0.000114
HYAL1	4.96E-05
ACAT1	4.50E-06
ADH6	0.001803
EPHX2	2.60E-05
GOT1	0.000649
ETNK2	0.000179
G6PC	0
PLOD2	0.001599
ACOX2	0.02195
RGN	0.039127
PLCG2	0.016466
PCK1	1.55E-05
PAH	0.004396
CHDH	0.000218
PTGDS	0.00236
CYP1B1	0.00984
CA4	0.000358
CRYL1	0
RDH12	0.022127
LCAT	0.004081
LPIN3	0.001354
KL	0
HAO2	1.55E-06
GATM	0.000178
AKR7A3	0.000202
PTGES	0.002517
MAOA	0.003098
AGMAT	0.000536
ACSM5	0.003401
CYP4A22	0.000733
ALDH1L1	5.61E-05
AOX1	9.85E-05
CRABP2	1.80E-07
ENPP2	0.03243
CYP4A11	0.001423
MIOX	9.45E-05
AOC1	0.004642

**Table 2:** Clinicopathological features between clusters in The Cancer Genome Atlas cohort

Variables	Cluster 1 (N = 217)	Cluster 2 (N = 309)	Overall (N = 526)
Stage			
Stage I	84 (38.7%)	179 (57.9%)	263 (50.0%)
Stage II	25 (11.5%)	31 (10.0%)	56 (10.6%)
Stage III	59 (27.2%)	63 (20.4%)	122 (23.2%)
Stage IV	48 (22.1%)	34 (11.0%)	82 (15.6%)
Missing	1 (0.5%)	2 (0.6%)	3 (0.6%)
Prior malignancy			
Clear cell adenocarcinoma, NOS	214 (98.6%)	298 (96.4%)	512 (97.3%)
Renal cell carcinoma, NOS	3 (1.4%)	11 (3.6%)	14 (2.7%)
Diagnosis			
no	190 (87.6%)	264 (85.4%)	454 (86.3%)
yes	27 (12.4%)	45 (14.6%)	72 (13.7%)
T			
T1	88 (40.6%)	181 (58.6%)	269 (51.1%)
T2	33 (15.2%)	35 (11.3%)	68 (12.9%)
T3	87 (40.1%)	91 (29.4%)	178 (33.8%)
T4	9 (4.1%)	2 (0.6%)	11 (2.1%)
N			
N0	104 (47.9%)	135 (43.7%)	239 (45.4%)
N1	12 (5.5%)	4 (1.3%)	16 (3.0%)
NX	101 (46.5%)	170 (55.0%)	271 (51.5%)
M			
M0	161 (74.2%)	255 (82.5%)	416 (79.1%)
M1	45 (20.7%)	33 (10.7%)	78 (14.8%)
MX	9 (4.1%)	21 (6.8%)	30 (5.7%)
Missing	2 (0.9%)	0 (0%)	2 (0.4%)
Gender			
female	56 (25.8%)	128 (41.4%)	184 (35.0%)
male	161 (74.2%)	181 (58.6%)	342 (65.0%)
Race			
asian	4 (1.8%)	4 (1.3%)	8 (1.5%)
black or african american	21 (9.7%)	35 (11.3%)	56 (10.6%)
not reported	1 (0.5%)	6 (1.9%)	7 (1.3%)
white	191 (88.0%)	264 (85.4%)	455 (86.5%)
Age			
> 60	110 (50.7%)	155 (50.2%)	265 (50.4%)
≤ 60	107 (49.3%)	154 (49.8%)	261 (49.6%)
Pharmaceutical			
no	64 (29.5%)	125 (40.5%)	189 (35.9%)
not reported	102 (47.0%)	154 (49.8%)	256 (48.7%)
yes	51 (23.5%)	30 (9.7%)	81 (15.4%)
Radiation			
no	82 (37.8%)	136 (44.0%)	218 (41.4%)
not reported	102 (47.0%)	150 (48.5%)	252 (47.9%)
yes	33 (15.2%)	23 (7.4%)	56 (10.6%)

### Characterization of the tumor microenvironment (TME) infiltrating the metabolism-related subclusters

**Fig. 4:**
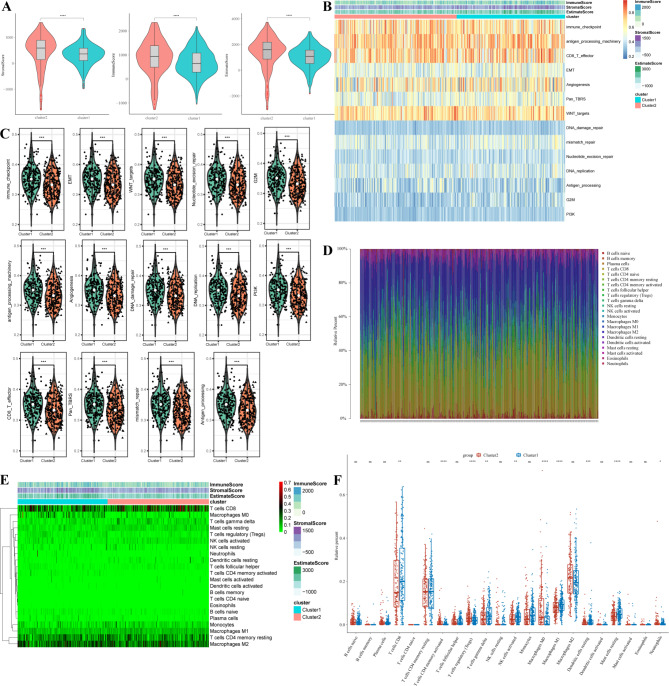
**Characterization of the tumor microenvironment (TME) infiltrating the metabolism-related subclusters** (**A**) Patterns of the stromal score, immune score, and Estimate score between metabolic-related subtypes. (**B**)-(**C**) Heatmap and violin plots showing the TME-related pathways. (**D**) Landscape showing the immune cell infiltration in the metabolic-related subclusters. (**E**) Heatmap showing the distribution of immune cell infiltration in the metabolic-related subclusters. (**F**) The abundance of 22 immune cells between metabolic-related subclusters using ssGSEA

We further investigated the TME characteristics in subclusters, ESTIMATE algorithm results indicated that the stromal score, immune score, and ESTIMATE score of cluster 2 higher than cluster 1, suggesting immune activation characteristics in cluster 2 (Fig. 4A). Then, we explored the differences of TEM-related pathways between subclusters, the results showed that immune checkpoint, epithelial-mesenchymal transition (EMT), WNT targets, nucleotide excision repair, G2M, antigen processing machinery, angiogenesis, DNA damage repair, DNA replication, PI3K, CD8 T effector, Pan TBRS, mismatch repair, antigen processing pathways were more enriched in cluster 1 (Fig. 4B-C). These data suggested complex biological processes in cluster 1. Then, we compared the fraction of immune cells between two subclusters, and the results showed that CD8 T cells, gamma delta T cells, activated NK cells, macrophages M1, resting dendritic cells (DCs), resting mast cells were abundant in cluster 1, while memory activated CD4 T cells, T cells follicular helper (Tregs), Macrophage M0, neutrophils were increased in cluster 2 (Fig. 4D-F). These results suggested that cluster 1 trended toward stromal and immune activation patterns, and cluster 2 was associated with the immunosuppressive phenotype.

### Correlation of the immunotherapy response and metabolism-related subclusters

**Fig. 5:**
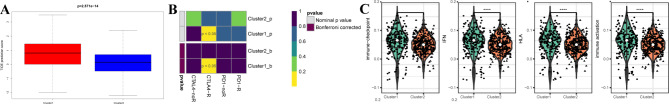
**Correlation of the immunotherapy response and metabolism-related subclusters** (**A**) Boxplot indicated the differences in TIDE score between two subclusters. (**B**) Submap showing the response to anti-PD1 and anti-CTLA4 treatment between two subclusters. (**C**) Violin plots showing the correlation between metabolic-related subclusters and immune checkpoint molecules

Considering the differences between the two subclusters, we investigated the different responsiveness of two subclusters to immune checkpoint blockade (ICB) therapy. The results showed a significantly higher TIDE score of cluster 1 than cluster 2 (Fig. 5A). And the submap results indicated the patients of cluster 1 respond to anti-CTLA4 treatment (Fig. 5B). Moreover, the GSVA results also supported that immune activation in cluster 1 than cluster 2 via immune checkpoint, IFN, HLA, and immune activation pathways (Fig. 5C). This indicated the significant differences in response to immunotherapy in two subclusters.

### Correlation of the chemotherapy response and metabolism-related subclusters

**Fig. 6:**
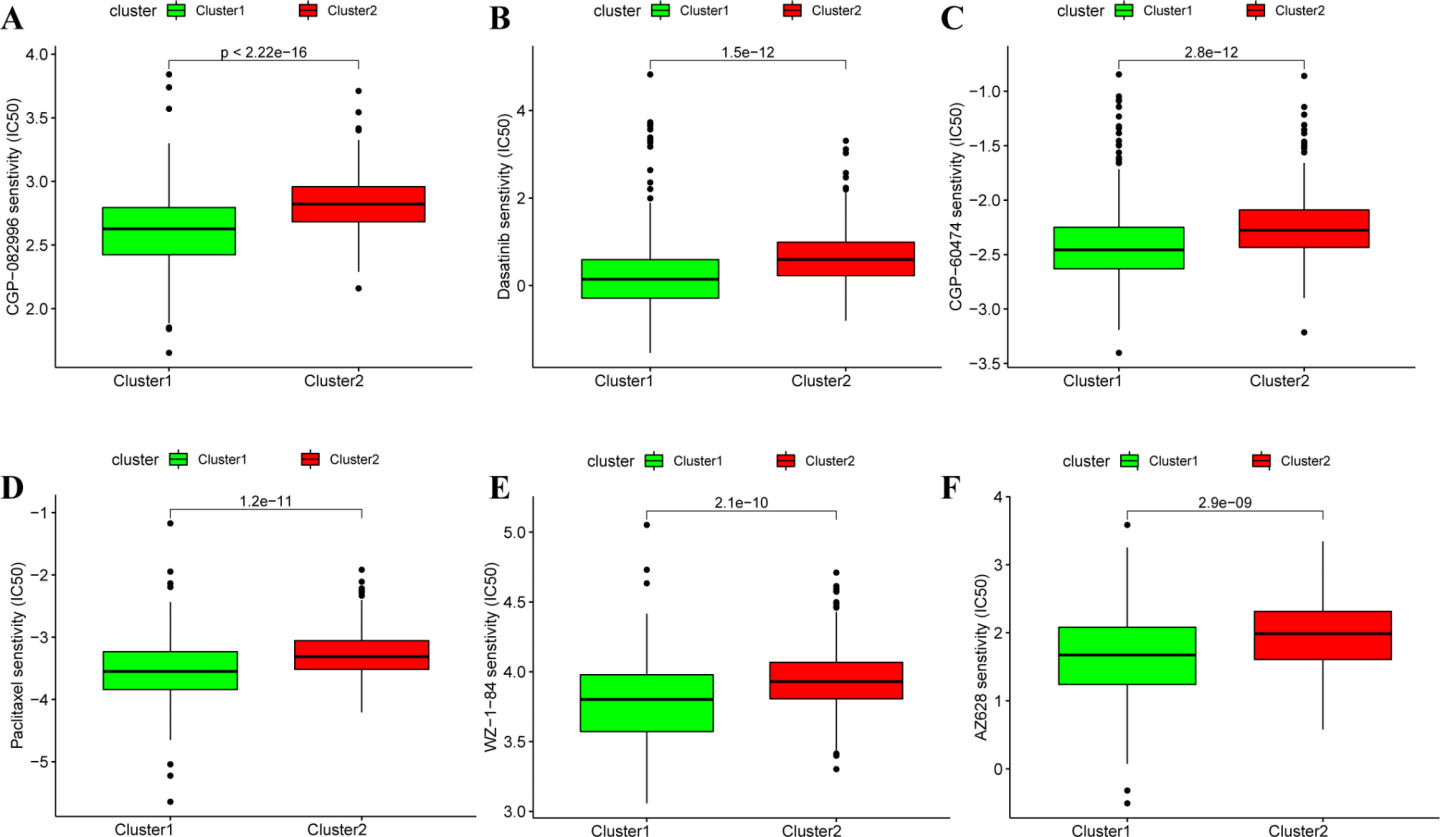
**Correlation of the chemotherapy response and metabolism-related subclusters** Boxplots depicted the differences in the IC50 values of (**A**) CGP-082996, (**B**) dasatinib, (**C**) CGP-60,474, (**D**) Paclitaxel, (**E**) WZ-1-84, (**F**) AZ628 between two subclusters

Here, we also explored the differences in the chemotherapeutic sensitivity between the two subclusters. Based on the GDSC database, we screened the sensitivity between the two subclusters to 138 common chemotherapeutic drugs, the results were shown in Fig. 6, IC50 values of CGP-082996, Dasatinib, CGP-60,474, Paclitaxel, WZ-1-84, and AZ628 for cluster 1 less than cluster 2. These data indicated that the patients in cluster 1 were more sensitive to CGP-082996, Dasatinib, CGP-60,474, Paclitaxel, WZ-1-84, and AZ628 than those in cluster 2.

### Identification of the prognostic signature between metabolism-related subclusters

**Fig. 7:**
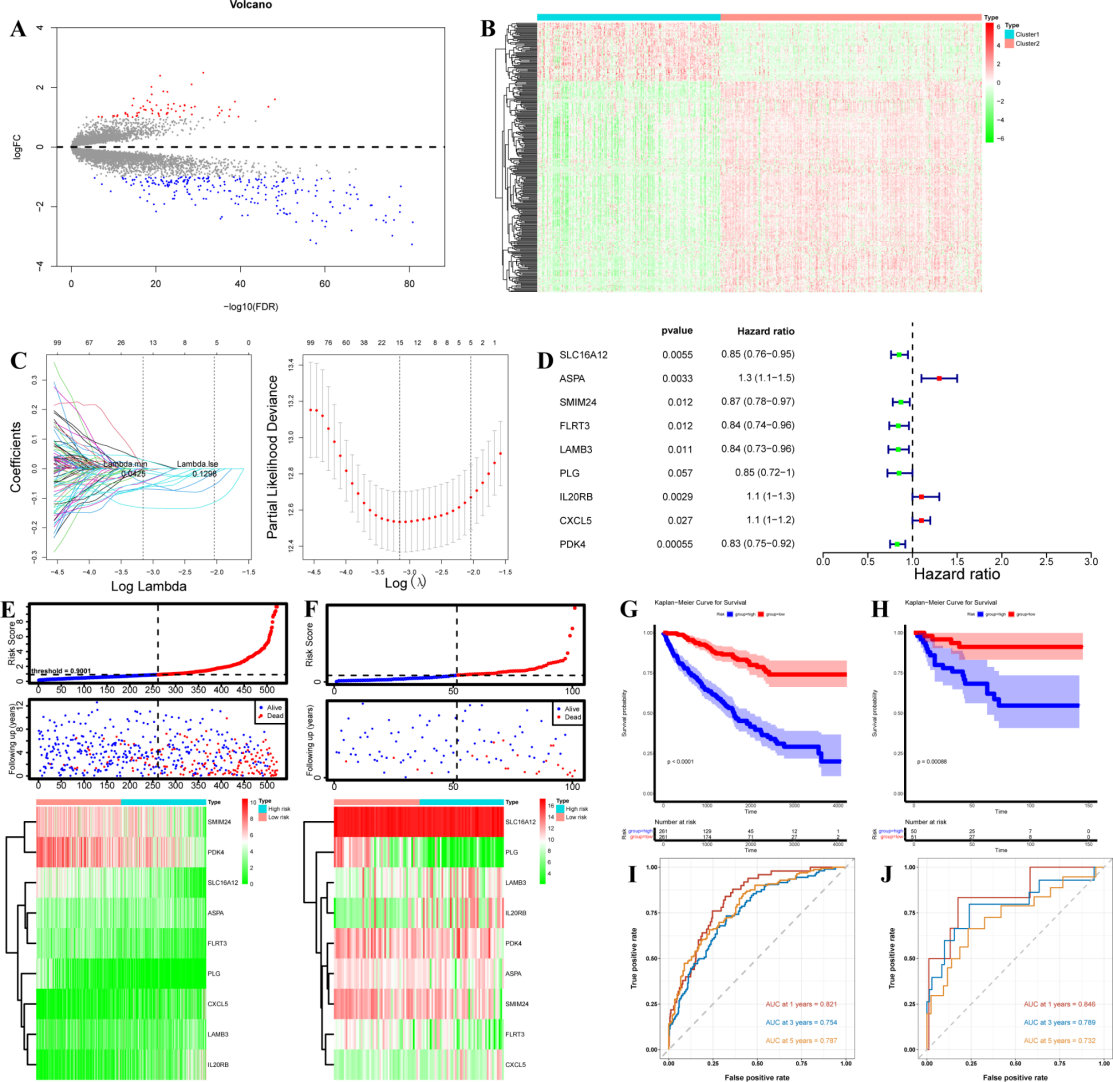
**Identification of the prognostic signature between metabolism-related subclusters** (**A**) Volcano plot showing the DEGs between two subclusters. (**B**) Heatmap showing the DEGs between two subclusters. (**C**) Left: Distribution of the coefficients of 15 genes at the optimal λ (grey line) for two subclusters. Right: LASSO regression model with 10-cross validation for selecting parameter that the optimal λ (dash line) which shows the minimum mean square error (red dots). (**D**) Forest plot indicated the nine risk factors identified by multivariable Cox regression analysis. (**E**)-(**F**) The risk score rank (up), the survival status (middle), and the expression of nine risk factors (bottom) between the high- and low-risk groups in TCGA-KIRC (training set) and E-MTAB-1980 (validation set) datasets. (**G**)-(**H**) KM OS curves for high- and low-risk groups in TCGA-KIRC and E-MTAB-1980 datasets. (**I**)-(**J**) Time-dependent ROC curves in 1-, 3-, 5-year OS time for high- and low-risk groups in TCGA-KIRC and E-MTAB-1980 datasets

Previous data indicated the heterogeneity of each sample and discrimination of response for antitumor therapy. Thus, we further investigated the prognostic risk signature between metabolism-related subclusters. First of all, we screened 295 DEGs between two metabolism-related subclusters (Table S3, Fig. 7A-B). With univariable cox analysis, 279 prognostic DEGs were identified between two subclusters (Figure S3, Table S4). Under LASSO cox regression analysis, 15 significant prognostic genes were identified between two subclusters (Fig. 7C). Then, a 15-gene signature was constructed, including ANK3, WDR72, PLS1, SLC16A12, ASPA, EMX2, SMIM24, EMCN, FLRT3, LAMB3, PLG, IL20RB, MDK, CXCL5, PDK4 (Table S5). Those genes were incorporated into a multivariable cox model to identify the independent risk factors, and 9 genes, including SLC16A12, ASPA, SMIM24, FLRT3, LAMB3, PLG, IL20RB, CXCL5, PDK4, were identified as independent risk factors for ccRCC (Fig. 7D, Table S6). The risk score of each sample was calculated based on risk factors, and the patients were distributed into high- and low-risk score groups according to the median risk score. The high-risk group showed a greater number of patient with dead than the low-risk group both in training and testing datasets (Fig. 7E-F). There was showed differential expression of risk factors between high- and low-risk groups in the training and testing datasets (Fig. 7E-F). As shown in Fig. 7G-H, the high-risk group showed the worse survival. To calculate the accuracy of the risk score for OS prediction using ROC curves, and the results showed that AUC for 1-, 3-, 5-years in the training set was 0.821, 0.754, and 0.787 (Fig. 7I). And the AUC for 1-, 3-, 5-years in the testing set was 0.846, 0.789, and 0.732 (Fig. 7J). These data indicated the good performance of this risk score for OS prediction.

#### Construction of the survival predictive nomogram in ccRCC

**Fig. 8:**
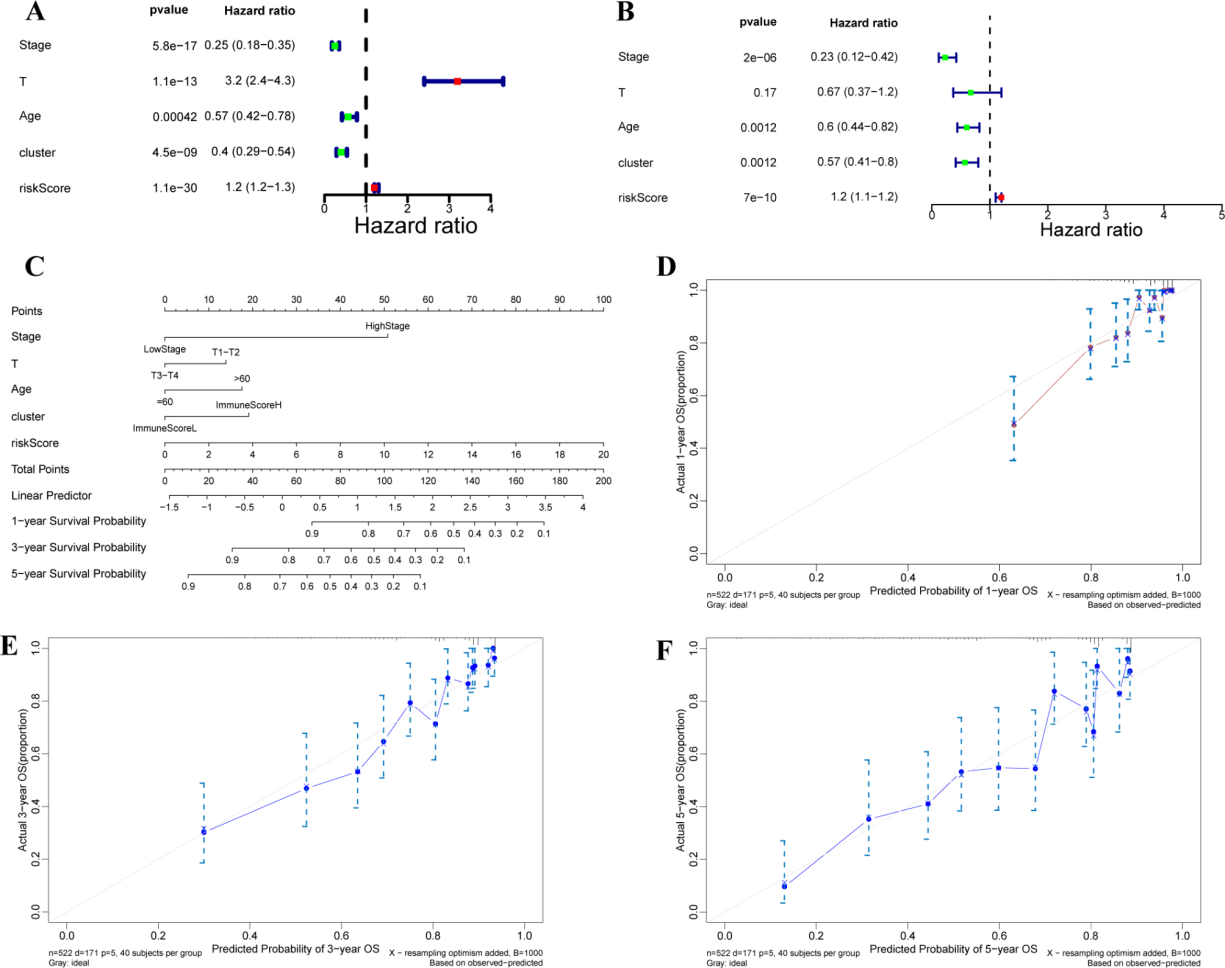
**Construction of the survival predictive nomogram in ccRCC** (**A**)-(**B**) Forest plots indicated the independent prognostic variables by incorporating the clinicopathological characteristics (age and stage), risk score, and molecular clustering. (**C**) A nomogram for 1-, 3-, 5-year OS prediction by combining independent prognostic variables. (**D**)-(**F**) Calibration plots indicated the performances of the nomogram-predicted probability of 1-, 3-, 5-year OS

We confirmed the molecular pattern, risk score, and clinical characteristics, including age and stage, were independent prognostic variables of the OS in the training set (Fig. 8A-B). We subsequently constructed a nomogram incorporating the molecular pattern, risk score, and clinical characteristics (age and stage) for predicting the OS of ccRCC patients (Fig. 8C). The calibration curves tested the predicted probability of 1-, 3-, and 5-years (Fig. 8D-F). These data suggested the nomogram integrating molecular pattern, risk score and clinical characteristics could boost the predictive efficiency of the prognosis of ccRCC patients.

## Discussion

Metabolism programming has become a central feature of ccRCC, which involves tumor initiation, progression, resistance to antitumor treatment, and poor survival rates in ccRCC patients [23–25]. In the present study, we comprehensively analyzed the metabolism-related molecular pattern and their characteristics in ccRCC. We identified the differentially expressed 254 MRGs between ccRCC tumor samples and non-tumor samples. Based on the differentially expressed 254 MRGs, 526 ccRCC patients from the TCGA database were clustered into two clusters and the clustering was verified by t-SNE and the GSE73731 dataset. Then, we investigated the biological function, molecular characteristics, TME infiltration feature, responsiveness to ICB and target therapy, and prognostic values between two subclusters.

Biological function and molecular characteristics analyses indicated that cluster 2 showed a poor survival rate and was associated with beta-alanine metabolism, fatty acid metabolism, glycerolipid metabolism, histidine metabolism, peroxisome, and PPAR signaling pathway, and starch and sucrose metabolism. Beta-alanine is not an essential amino acid and exerts as a sports supplement to increase anaerobic endurance and athletic performance, beta-alanine can be used as a potential antitumor agent in malignant breast epithelial cells, renal tumor cells, and cervical tumor cells [26, 27]. Another common and important amino acid metabolism is histidine metabolism, histidine catabolism increases the effectiveness of methotrexate treatment in cancers [28]. Serum histidine level is a potential predictive biomarker for patients with ccRCC [29, 30]. Fatty acid and its decomposition product glycerolipid metabolism are associated with tumor progression [31, 32]. The peroxisome is a metabolic organelle involved in lipid metabolism and cellular redox balance [33], regulation of the peroxisome proliferator-activated receptor (PPAR) contributes to cellular homeostasis by feedback regulation of the expression of enzymes that involve glucose, amino acid, and lipid metabolism [34]. Low levels of PPAR are associated with poor clinical outcomes in hepatocellular carcinoma (HCC) and ccRCC patients [35]. The above shreds of evidence have demonstrated cluster 2 is associated with complex metabolic processes, including lipid, amino acid, and glucose metabolism.

We also investigated the TME infiltration feature of two subclusters. The stromal score, immune score, and ESTIMATE score of cluster 2 were higher than in cluster (1) at CD8 T cells, gamma delta T cells, activated NK cells, macrophages M1, resting DCs, resting mast cells were abundant in cluster 1, while memory activated CD4 T cells, Tregs, Macrophage M0, neutrophils were increased in cluster (2) Furthermore, we found the stromal activation associated with biological processes, such as EMT, WNT targets, angiogenesis, and Pan TBRS significantly enriched in cluster 1 than in cluster 2. And the effector immune cells, such as CD8 T cells [36], and activated NK cells [37], were abundant in cluster (1) The gamma delta T cells represent a small population that performs complex immune regulatory functions and promotes tumor progression [38, 39], and exhibit the potential for cancer immunotherapy [40, 41]. The above finding indicated cluster 1 might sensitive to ICB therapy, and cluster 2 might poorly respond to ICB therapy. Consistent with those finding and speculation, cluster 1 with higher TIDE score and respond to anti-CTLA4 treatment, and cluster 2 couldn’t. Besides, immune checkpoint, IFN, HLA, and immune activation pathways enriched in cluster 1 than cluster 2, suggesting immune activation phenotype in cluster 1, and cluster 1 showed sensitivity to ICB therapy. Moreover, we also found cluster 1 more sensitive to CGP-082996, dasatinib, CGP-60,474, Paclitaxel, WZ-1-84, and AZ628 than those in cluster (2) Dasatinib is an orally multi-target kinase inhibitor that emerges the antitumor effects in RCC patients by suppressing tumor cell proliferation [42]. Paclitaxel is a first-line treatment for some tumors [43–45], and it is in combination with other chemotherapy drugs for the treatment of RCC patients [46, 47].

Previous sections analyzed the biological function, molecular characteristics, TME infiltration feature, responsiveness to ICB, and target therapy of molecular subclusters. Here, we further investigated the prognostic values of metabolism-related clustering. We identified the 295 DEGs between two subclusters. And a 15-gene signature was constructed, including ANK3, WDR72, PLS1, SLC16A12, ASPA, EMX2, SMIM24, EMCN, FLRT3, LAMB3, PLG, IL20RB, MDK, CXCL5, PDK4. Then, SLC16A12, ASPA, SMIM24, FLRT3, LAMB3, PLG, IL20RB, CXCL5, and PDK4 were identified as risk factors for ccRCC patients. The prognostic values of those risk factors were verified by ROC curves and previous studies. Such as, SLC16A12 is a creatine transporter for creatine and guanidinoacetate in the kidney [48], and its expression level predicates a favorable prognosis for ccRCC patients [49]. LAMB3 is a common oncogene in tumors [50, 51], but its role and function of it remain undiscovered. Increasing IL20RB expression associates tumor progression and poor prognosis in papillary RCC [52], and relates to poor survival for ccRCC patients [53]. CXCL5 cytokine promotes RCC progression and can be used as the therapeutic target for RCC treatment [54, 55]. PDK4 is a metabolism gene that promotes tumor development [56, 57], and acts as a prognostic biomarker in ccRCC [58].

## Conclusion

In conclusion, a metabolism-related molecular pattern for ccRCC was constructed, and we also investigated the biological function, molecular characteristics, TME infiltration feature, responsiveness to ICB and target therapy, and prognostic values between two subclusters. Based on the differences, a prognostic signature and a risk model were constructed for survival predicting in ccRCC. Our finding suppled a novel insight for ccRCC diagnosis and prognosis prediction. However, more experimental evidence is needed to validated in a larger internal cohort, and the function of these MAGs in cellular phenotypes will also be discussed.

### Electronic supplementary material

Below is the link to the electronic supplementary material.

Supplementary Material 1

Supplementary Material 2

Supplementary Material 3

Supplementary Material 4

Supplementary Material 5

Supplementary Material 6

Supplementary Material 7

Supplementary Material 8

Supplementary Material 9

## Data Availability

The data used in this study are freely available from TCGA, GEO, and ArrayExpress databases.
