# Supplementary figures and images for: Construction of the metabolic reprogramming-associated gene signature for clear cell renal cell carcinoma prognosis prediction
Source: BMC Urol. 2023 Sep 15;23:147. doi: 10.1186/s12894-023-01317-3 (PMC10503121; doi:10.1186/s12894-023-01317-3)

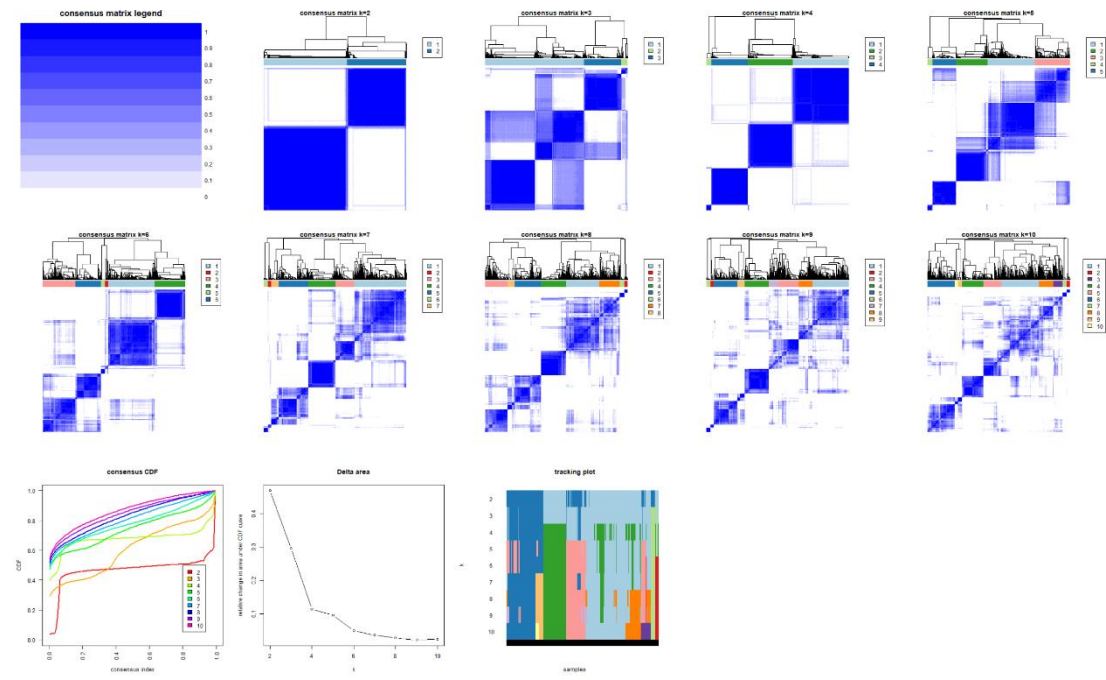

**Figure S1 Consensus clustering of 526 ccRCC patients in TCGA-KIRC dataset.**

Supplement: Supplementary file 7 — Supplementary Material 7 [file 12894_2023_1317_MOESM7_ESM.pdf]

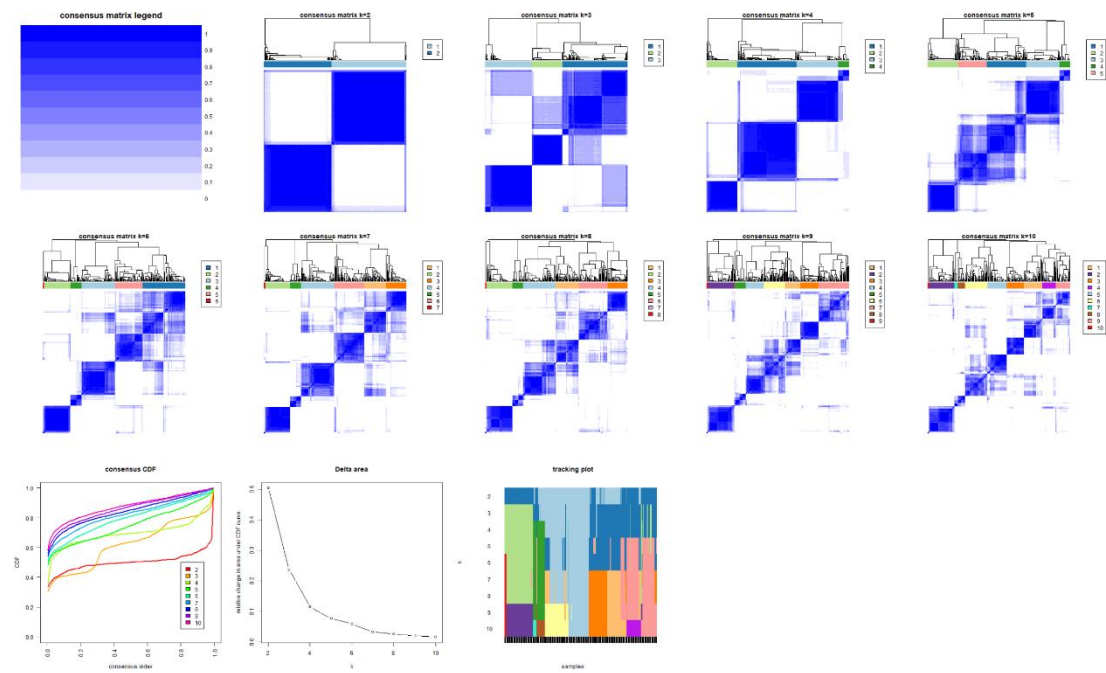

**Figure S2 Consensus clustering of 265 ccRCC patients in the GSE73731 dataset.**

Supplement: Supplementary file 8 — Supplementary Material 8 [file 12894_2023_1317_MOESM8_ESM.pdf]

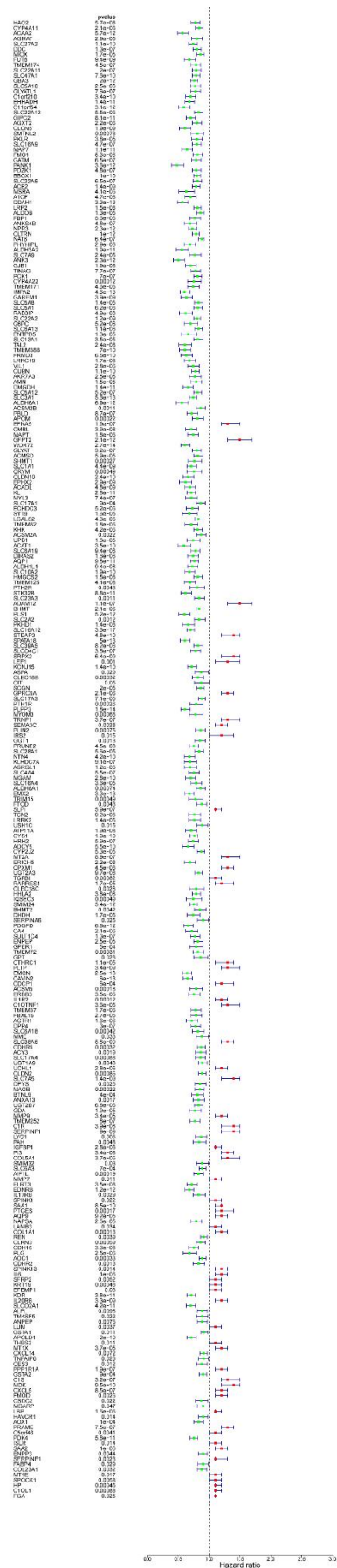

**Figure S3 Forest plot of 279 prognostic DEGs between two subclusters.**

Supplement: Supplementary file 9 — Supplementary Material 9 [file 12894_2023_1317_MOESM9_ESM.pdf]
